# Phosphatidylethanolamine aggravates Angiotensin II-induced atrial fibrosis by triggering ferroptosis in mice

**DOI:** 10.3389/fphar.2023.1148410

**Published:** 2023-05-23

**Authors:** Fangze Huang, Ximao Liu, Junjie Liu, Yu Xie, Li Zhao, Deshen Liu, Zifeng Zeng, Xiu Liu, Shaoyi Zheng, Zezhou Xiao

**Affiliations:** Department of Cardiovascular Surgery, Nanfang Hospital, Southern Medical University, Guangzhou, China

**Keywords:** atrial fibrillation, atrial fibrosis, lipidomics, phosphatidylethanolamine, ferroptosis, peroxidation, cardiovascular diseases

## Abstract

As atrial fibrosis is the main feature of atrial structural remodeling, inhibiting atrial fibrosis is crucial to the prevention of atrial fibrillation (AF) progression. Research has shown the correlation between abnormal lipid metabolism and AF progression. However, the effect of specific lipids on atrial fibrosis remains unclear. In the present study, we applied ultra-high-performance lipidomics to analyze the lipid profiles in patients with AF and identify phosphatidylethanolamine (PE) as the differential lipid associated with AF. To detect the effect of the differential lipid on atrial fibrosis, we performed the intraperitoneal injection of Angiotensin II (Ang II) to mice to induce atrial fibrosis and supplemented PE in diets. We also treated atrial cells with PE to evaluate the cellular effect of PE. We found that PE supplementation aggravated atrial fibrosis and increased the expression of the fibrosis-related protein *in vitro* and *in vivo*. Moreover, we detected the effect of PE on the atrium. We found that PE increased oxidation products and regulated the expression of ferroptosis-related proteins, which could be alleviated by a ferroptosis inhibitor. PE increased peroxidation and mitochondrial damage *in vitro*, which promoted cardiomyocyte death induced by Ang II. Examination of protein expression in cardiomyocytes indicated that PE triggered ferroptosis and caused cell death to participate in myocardium fibrosis. In summary, our findings demonstrated the differential lipid profiles of AF patients and revealed the potential effect of PE on atrial remodelling, suggesting that inhibition of PE and ferroptosis might serve as a potential therapy to prevent AF progression.

## 1 Introduction

Atrial fibrillation (AF) is one of the most common cardiac arrhythmias in the global population, which is correlated with high morbidity, high mortality, and severe clinical burden (Dai et al., 2021). It presents as an irregular heartbeat, which is caused by rapid and chaotic electrical signals in the atria (Brundel et al., 2022). With AF related to adverse health outcomes and high healthcare costs, preventing the occurrence and progression of AF is urgently needed. Atrial fibrosis is a major factor for cardiac remodeling in AF, in which connective myocardium is redistributed and replaced with pathological tissue (Nattel, 2017). It is a common characteristic in patients with AF, which destroys cellular connections of cardiomyocytes by disturbing the mechanical transduction of electricity (Reese-Petersen et al., 2020). As a result, atrial fibrosis is the main feature of atrial structural remodeling, which is significant for the initiation and maintenance of AF (Pellman and Sheikh, 2015). It has been reported that the renin-angiotensin system (RAS) takes part in the development of atrial fibrosis with Angiotensin II (Ang II) as the effector (Li et al., 2019). In fact, Ang II exerts pro-fibrotic effects in various ways such as increasing Ca^2+^ levels, promoting proliferation and differentiation of fibroblast, and overproducing reactive oxygen species (ROS) (Li C. Y. et al., 2021). Thus, Ang II has been widely used in research to induce atrial fibrosis for further learning of the mechanism of atrial remodeling in AF (Lu et al., 2021; Xiao et al., 2021).

Abnormal lipid metabolism is the main risk factor for AF, as metabolic and lipidomic transformation might cause atrial remodeling (Suffee et al., 2021). Aiming to discover the correlation between lipid metabolism and AF, many researchers have applied lipidomics to identify global changes in lipid metabolites in AF. In 2019, a study used lipidomics to analyze lipid changes in patients with AF and discovered the upregulation of free fatty acids was associated with AF (Zhou et al., 2019). Them et al. studied a cohort of 3,779 patients with lipidomics and revealed the correlation between phospholipids and AF prevalence, especially with specific phosphatidylethanolamine (PE) (Tham et al., 2020).

PE is the second amplest phospholipid in mammalian cells, which is the major component of biomembrane and abundant in mitochondrial membrane (Patel and Witt, 2017). Recently, PE has been reported to be correlated with cardiovascular disease. In a prospective population-based Bruneck Study which is a survey of atherosclerosis and cardiovascular diseases in 2000, PE was demonstrated to be associated with cardiovascular diseases with PE (36:5) possessing the strongest predictive value (Stegemann et al., 2014). Moreover, the content of PE affects oxidative phosphorylation and mitochondrial function, which is implicated in cardiovascular diseases (Tasseva et al., 2013). As the ethanolamine part of PE can be covalently modified in various ways, PE has multiple functions in cells. In fact, PE has been reported to associate with various cell functions including programmed cell death (PCD) (Kagan et al., 2017), autophagy (Ichimura et al., 2000), and mitochondrial fusion (Verkleij et al., 1984). In particular, the correlation between PE and PCD is mainly presented as the induction of PE on ferroptosis, an iron-dependent cell death (Dixon et al., 2012; Kagan et al., 2017). Ferroptosis is a process of iron-related accumulation of lethal lipid ROS that devastates the integrity of cell membranes (Qiu et al., 2020). In ferroptosis, phospholipid peroxidation is the primary driving factor, in which glutathione (GSH) is depleted with the deactivation of glutathione peroxidase 4 (GPX4) (Dixon et al., 2012; Yang et al., 2014). As the hallmark of ferroptosis, lipid peroxidation leads to lipid peroxides attacking polyunsaturated fatty acyl moieties present on phospholipids, which can devastate membrane integrity (Jiang et al., 2021). Therefore, ferroptosis is closely associated with the process of lipid synthesis, storage, and degradation. Moreover, lipid metabolism can regulate ferroptosis and determine the sensitivity of cells to ferroptosis. Tesfay et al. demonstrated that decreasing CoQ10 and unsaturated fatty acyl chains in membrane phospholipids in monounsaturated fatty-acid synthesis could induce ferroptosis (Tesfay et al., 2019). Also, it is reported that PE with arachidonic acid (AA) or adrenic acid (AdA) is the signal to actuate ferroptosis (Kagan et al., 2017), which is catalyzed by acyl-CoA synthetase long-chain family member 4 (ACSL4) to restructure cell membrane and promote the lipid oxidization (Dixon et al., 2015; Xie et al., 2016).

Recently, some research has proved that ferroptosis is involved in the pathogenesis of cardiovascular diseases. Ferroptosis is observed in various cardiovascular pathological models such as myocardial infarction, myocardial ischemia/reperfusion injury, heart failure, diabetic cardiomyopathy, and so on (Wu et al., 2021). The application of specific ferroptosis inhibitors including ferrostatin-1 (Fer-1) and dexrazoxane ameliorates the effect of ferroptosis on cardiomyocyte death (Fang et al., 2019). Also, it has been reported that iron levels were correlated with severe cardiac injury and hypertrophic cardiomyopathy. Though a recent study has reported the potential role of ferroptosis in hypertension induced by Ang II (Zhang Z. et al., 2022), the relationship between ferroptosis and Ang II-induced atrial fibrosis remains to be explored. In this study, we aimed to discover the effect of PE on the atrial tissues induced by Ang II and to investigate whether ferroptosis was triggered by PE in cardiomyocytes.

## 2 Materials and methods

### 2.1 Patients

From 2022 June to 2022 August, we recruited 12 participants in this study. It was composed of 6 healthy controls and 6 AFs, with an electrocardiogram (ECM) or 24-hour dynamic electrocardiogram (DCG) indicating sinus rhythm (SR) or AF. AFs group consisted of patients who underwent elective cardiac valve replacement at the Department of Cardiac Surgery, Southern Medical University Nanfang Hospital, Guangzhou, China. Patients suffering from cancer, heart failure, stroke, peripheral artery disease, and infective endocarditis were excluded. Healthy volunteers admitted no history of diabetes mellitus or hypercholesterolemia. Individuals were also excluded if they had received medications with hypolipidemic effects. This study was approved by the Ethics Committee of Nanfang Hospital, Southern Medical University (approval number NFEC-2022–391) and implemented in accordance with the Declaration of Helsinki. All patients were informed and agreed to participate with formal consent obtained.

### 2.2 Blood sample collection and lipid extraction

Blood samples were collected preoperatively in the AF group, while controls had their blood drawn when limosis. Samples were centrifuged at 3,000 rpm for 10 min at room temperature to obtain blood plasma, which was stored at −80°C. Samples were first spiked with an appropriate amount of internal lipid standards and then homogenized with 200 µL water and 240 µL methanol. After that, 800 µL of methyl tert-butyl ether was added and the mixture was sonicated for 20 min at 4°C followed by standing at room temperature for 30 min. The solution was centrifuged at 14,000 g for 15 min at 10°C and the upper organic solvent layer was obtained and dried under nitrogen.

### 2.3 Ultra-high-performance liquid chromatography-tandem mass spectrometry (UHPLC-MS/MS) method for lipid analysis

Reverse phase chromatography was selected for LC separation using CSH C18 column (1.7 µm, 2.1 mm × 100 mm, Waters). The lipid extracts were re-dissolved in 200 µL 90% isopropanol/acetonitrile and centrifuged at 14,000 g for 15 min. Finally, 3 µL of the sample was injected. Solvent A was acetonitrile-water (6:4, v/v) with 0.1% formic acid and 0.1 Mm ammonium formate and solvent B was acetonitrile–isopropanol (1:9, v/v) with 0.1% formic acid and 0.1 Mm ammonium formate. The initial mobile phase was 30% solvent B at a flow rate of 300 μL/min. It was held for 2 min, and then linearly increased to 100% solvent B in 23 min, followed by equilibrating at 5% solvent B for 10 min. Mass spectra were acquired by Q-Exactive Plus in the positive and negative modes, respectively. Electron spray ionization (ESI) parameters were optimized and preset for all measurements as follows: Source temperature, 300°C; Capillary Temp, 350°C, the ion spray voltage was set at 3,000 V, S-Lens RF Level was set at 50% and the scan range of the instruments was set at m/z 200–1800.

### 2.4 Experimental animals

C57BL/6J mice and Sprague-Dawley rats in the study were obtained from the Laboratory Animal Center of Southern Medical University. Mice were raised in a standard animal facility, with a room temperature of 21°C–24°C and a 12-h light/dark cycle. The study was approved by the Animal Research Committee of Center of Nanfang Hospital and conducted according to the recommendations of the Guide for the Care and Use of Laboratory Animals (NIH, 8th edition, 2011).

### 2.5 Animal treatments and grouping

Male C57BL/6 mice (6–8 weeks) were randomly assigned into sham group, Ang II group, PE group, Ang II + PE group, Ang II + Fer-1 group, PE + Fer-1 group, and Ang II + PE + Fer-1 group. Mice were accepted 2 mg/kg/d Ang II (Aladdin, Shanghai, China), 1 mg/kg/d Fer-1 (Selleck, Shanghai, China), and equal normal saline (containing 0.1% DMSO) by intraperitoneal injection for 4 weeks according to the different assignment. The Ang II and Fer-1 were dissolved in DMSO before being diluted with normal saline, with a DMSO concentration of 0.1%. For the PE-induced model, mice were fed normal fodder and water with 0.2% PE (Aladdin, Shanghai, China). After 4 weeks, mice were anesthetized by injecting 60 mg/kg pentobarbital sodium and 50 mg/kg ketamine to obtain the atrial tissues.

### 2.6 Masson staining

The atrial tissues were fixed in 4% paraformaldehyde (PFA) for 24 h to make paraffin sections in 4 μm. Then the paraffin sections were deparaffinized by being exposed to xylene and dehydrated by gradient ethanol. Next, the paraffin sections underwent the following steps: staining with ponceau-acid fuchsin solution for 5 min, being treated with phosphomolybdic acid solution for 3–5 min, and counterstain by toluidine blue-O for 30–60 s. Sections were immersed in 1% glacial acetic acid for 1 min before dehydration, permeabilization, and seal. Images were acquired with a standard microscope.

### 2.7 Immunohistochemistry (IHC)

For IHC assays, primary antibodies against *α*-smooth muscle actin (α-SMA) (1:200, 67735-1-Ig, Proteintech), GPX4 (1:200, ab125066, Abcam), ACSL4 (1:200, 22,401-1-ap, Proteintech) were used. Paraffin sections were subjected to antigen retrieval in citrate buffer for 20 min. After washing in phosphate-buffered saline (PBS) 5 min for 3 times, the sections were blocked in blocking/permeabilizing buffer for 1 h. Then sections were incubated with the primary antibody mentioned above at 4°C overnight. Avidin–biotin peroxidase detection systems with DAB substrate were used to mark the locations of antigens, followed by counterstaining with hematoxylin. Images were acquired with a standard microscope.

### 2.8 Cell culture and treatments

Newborn Sprague-Dawley rats were anesthetized by injecting pentobarbital sodium (60 mg/kg) and ketamine (50 mg/kg) before thoracotomy. The atrial tissues were isolated and digested with 0.2% pancreatin and collagenase II (1 mg/mL). After differential centrifugation and resuspension, atrial fibroblasts and cardiomyocytes were obtained. The cells were cultured in Dulbecco’s Modified Eagle Medium (DMEM) containing 10% fetal bovine serum (FBS), penicillin, and streptomycin (100:1, Gibco), and placed in a 5% CO_2_ incubator at 37 °C. Before being treated with drugs, the cells were starved by culturing for 12–24 h in DMEM without FBS. The cells were stimulated with 10 μM Ang II, 1 μM Fer-1, 1 μM erastin, and PE at various concentrations for 24 h.

### 2.9 Cell counting kit-8 (CCK-8) assay

Cardiomyocyte viability was detected by the standard CCK-8 kit according to the instruction. Cardiomyocytes were seeded in the 96-well plates and cultured for 24 h in the incubator. Then, 10 µL CCK-8 solution was added to each well and incubated for 3 h. The absorbance at 450 nm wavelength was measured with the standard microplate reader.

### 2.10 GSH level detection

GSH level was detected by the standard reduced GSH colorimetric assay kit (Elabscience, China) according to the instruction. Atrial tissues homogenate (10%, w/v) was centrifuged at 10,000 g at 4°C for 10 min and the supernatants were collected. Cardiomyocytes (1 × 10^6^/well) were sonicated with 300–500 μL homogenization medium. Homogenate was centrifuged at 1,500 g for 10 min to obtain supernatants. Supernatants were mixed with reagents at room temperature for 15 min. The absorbance at 420 nm was measured using a microplate reader.

### 2.11 Iron level detection

The iron level was detected by the standard ferrous iron colorimetric assay kit (Elabscience, China) according to the instruction. Atrial tissues were mixed with buffer solution (10%, w/v) and then centrifuged at 10,000 g for 10 min to collect supernatants. Cardiomyocytes (1 × 10^6^/well) were mixed with 200 μL buffer solution and centrifuged at 15,000 g at 4°C for 10 min. Supernatants were mixed with reagents and developer at 37°C for 10 min. The absorbance at 593 nm was measured using a microplate reader.

### 2.12 Malondialdehyde (MDA) assay

MDA level was assessed using the standard MDA colorimetric Assay Kit (Elabscience, China) according to the instruction. Atrial tissues were mixed with buffer solution (10%, w/v) and then centrifuged at 10,000 g for 10 min to collect supernatants. Cardiomyocytes (3 × 10^6^/well) were mixed with 500 μL extracting solution for 2 min and sonicated for 10 min. Supernatants were mixed with reagents and heated at 100°C for 40 min, followed by centrifugation at 3,100 g for 10 min. The absorbance at 532 nm was measured using a microplate reader.

### 2.13 NADPH/NADP^+^ ratio measurement

NADPH/NADP^+^ ratio was measured using the NADP^+^/NADPH Colorimetric Assay Kit (Elabscience, China) according to the instruction. Atrial tissues were mixed with buffer solution and centrifuged at 12,000 g at 4°C for 10 min to collect supernatants. Cardiomyocytes (4 × 10^6^/well) were mixed with 2–5 mL iced PBS and centrifuged at 1,000 g at 4 °C for 10 min. Supernatants were mixed with 800 μL iced extracting solution for 10 min, followed by centrifugation at 12,000 g at 4 °C for 10 min. Then, supernatants were mixed with reagents and cultured at 37 °C for 10 min. The absorbance at 450 nm was measured using a microplate reader.

### 2.14 Transmission electron microscopy (TEM)

Mitochondrial morphology was observed by TEM. Small cardiomyocyte fragments (1 mm^3^ in volume) were fixed in 2.5% glutaraldehyde at 4°C for 2–4 h followed by rinsing 15 min for 3 times in phosphate buffer (0.1 M, pH 7.4). Then the fragments were fixed in 1% osmium tetroxide phosphate buffer (0.1 M, pH 7.4) at 20 °C for 2 h. After rinsing in phosphate buffer, the fragments were dehydrated in a graded ethanol series (50, 70, 90, and 100%). Then the fragments were permeabilized with a 1:1 mixture of propylene oxide and Epon 812 for 2–4 h and with a 1:2 mixture of propylene oxide and Epon 812 overnight. After being baked at 37°C, the fragments were cut into ultrathin sections (80 nm) with an ultramicrotome (UC7, Leica, Germany). The sections were stained with 2% uranyl acetate and lead citrate and examined with an electron microscope (JEM-1400, Japan).

### 2.15 ROS detection assay

Intracellular ROS levels were detected by ROS Assay Kit (Beyotime Biotechnology, Shanghai, China). After different reagent treatments, the cells were incubated with the probe 2′,7′-dichlorofluorescin diacetate (DCFH-DA) (diluted 1:1,000) in a 37 °C incubator in the dark for 30 min. After washing with PBS three times, the fluorescence of the cells was imaged with an excitation of 488 nm/emission of 529 nm. The fluorescence intensity was measured using ImageJ_v1.8.0 software.

### 2.16 Immunofluorescence

Cells were fixed in 4% PFA at room temperature for 10 min and permeabilized with 0.1% Triton X-100 in PBS for 20 min at room temperature. Then cells were blocked for 30 min at 37°C and incubated with primary antibody at 4°C overnight. After washing with PBS, cells were incubated with secondary antibody for 2 h at room temperature. DAPI was used to stain nucleus. Images were acquired with a standard microscope.

### 2.17 Western blotting

Proteins were extracted from the cardiomyocytes and atrial tissues using RIPA lysis buffer (Beyotime Biotechnology, Shanghai, China) and quantified by BCA assay (Beyotime). Electrophoresis was set for 30 min at 80 V and then for 90 min at 120 V. Later, the proteins were transferred onto PVDF membranes (Millipore, United States). The membranes were blocked with 5% non-fat milk for 1 h at room temperature and then incubated with primary antibodies overnight at 4°C. The primary antibodies against *α*-SMA (1:1,000, 67735-1-Ig, Proteintech), GPX4 (1:1,000, ab125066, Abcam), ACSL4 (1:1,000, 22,401-1-ap, Proteintech), and GAPDH (1:10000, 10494-1-AP, Proteintech). After washing with TBST 3 times, the membranes were incubated with specific secondary antibodies for 1 h at room temperature. Signals were detected by the standard ECL kit (Biosharp, China). Densitometry was evaluated with ImageJ_v1.8.0 software.

### 2.18 Quantitative reverse transcription polymerase chain reaction (RT-qPCR)

Total RNA was extracted from the cardiomyocytes and atrial tissues by TRIzol (Ambion, United States). Reverse transcription of RNA samples to generate cDNA was performed with the reverse transcription reagent Evo M-MLV RT Master Mix (Agbio, Hunan, China), which was conducted as follows: 37°C for 15 min, 85°C for 5 s and hold at 4°C. Gene expression was detected by employing a LightCycler 480 real-time PCR instrument (Roche, Indianapolis, IN, United States). The reaction conditions were set according to the instructions of the fluorescent quantitative PCR kit (SYBR Green Premix, Agbio, Hunan, China). The results were analyzed with the 2^−△△Ct^ method and normalized to GAPDH gene expression. The primers are listed in Table 1.

**TABLE 1:** List of RT-PCR primers.

Gene	Sequence (5′–3′)
α-SMA-F	CCC​AGA​CAT​CAG​GGA​GTA​ATG​G
α-SMA-R	TCT​ATC​GGA​TAC​TTC​AGC​GTC​A
GPX4-F	ACG​AAT​TCT​CAG​CCA​AGG​ACA​T
GPX4-R	ATG​CAG​ATC​GAC​TAG​CTG​AGT​G
ACSL4-F	CGG​GAG​ATC​CTG​AGT​GAA​GAA​A
ACSL4-R	TGG​CAA​TGG​TGT​TCT​TTG​GTT​T
GAPDH-R	CAT​CAC​TGC​CAC​CCA​GAA​GAC​TG
GAPDH-F	ATG​CCA​GTG​AGC​TTC​CCG​TTC​AG

### 2.19 Statistical analyses

Quantitative data were presented as the mean ± standard deviation (SD). Comparisons among multiple groups were assessed by one-way analysis of variance (ANOVA) and confirmed by Tukey’s multiple comparisons test. A *p*-value <0.05 was regarded as statistically significant. All statistical analyses were performed using GraphPad Prism 8.0 software (San Diego, CA, United States).

## 3 Results

### 3.1 Lipid profiling indicates that PE level is higher in patients with AF

**FIGURE 1:**
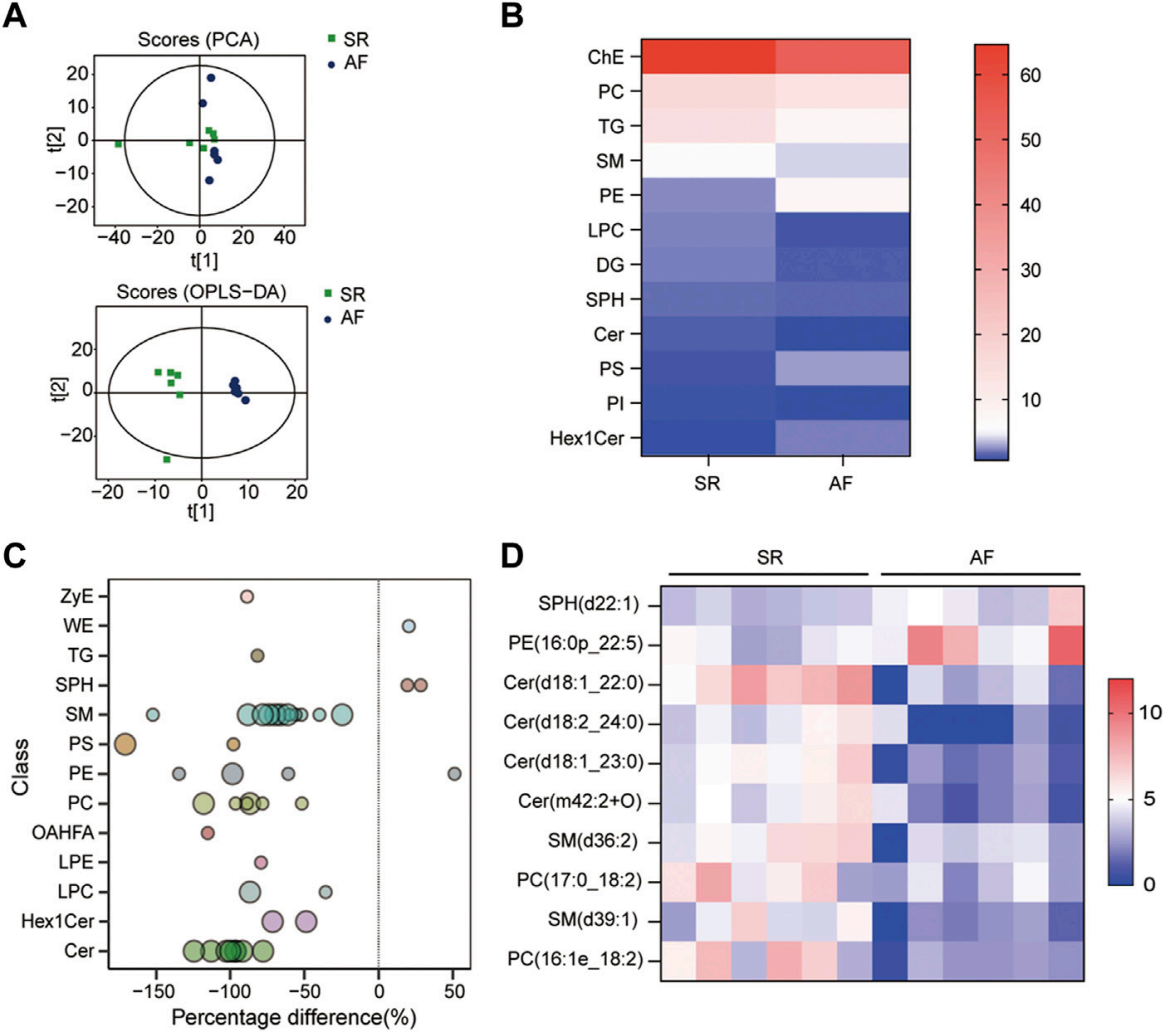
Lipid profiles in AF and SR groups. **(A)** Principle component analysis (PCA) and orthogonal partial least square analysis (OPLS-DA) score plots of lipids from SR and AF groups. **(B)** Lipidomic analyses of lipid classes (μg/mL) from serum samples in SR and AF groups. **(C)** Bubble plot of significantly differential lipids in SR and AF group. Differential lipid was identified as the variables with variable importance for the projection (VIP) of OPLS-DA >1 and *p*-value < 0.05. Bubbles in the plot represent the significantly differential lipids. The vertical axis represents the lipid subclass, discriminated by different colors. The size of bubbles represents the significance of difference, with smaller bubbles as *p*-value between 0.01 and 0.05, and larger bubbles as *p*-value < 0.01. **(D)** Lipidomic analyses of differential lipid species (μg/mL) from serum samples in SR and AF groups.

To identify the difference in lipid metabolism between healthy controls and patients with AF, we collected serum samples from 12 individuals with 6 healthy controls who presented sinus rhythm in ECM or DCG and 6 AF patients. Baseline characteristics were presented in Table 2. All subjects in the AF group were first diagnosed with AF prior to any antiarrhythmic treatment. Serum samples from each participant were disposed to extract lipid, which was analyzed by absolute quantitative lipidomics based on UHPLC-MS/MS. A total number of 34 lipid classes and 2,576 lipid species were determined in the SR and AF group. The concentration of total lipids in the AF group was higher compared to that in the SR group (5,036.13 vs. 703.60 μg/mL; *p* = 0.67). To distinguish the lipid profiling between the two groups, we performed principal component analysis (PCA) to remove outlier samples and visualize data. To further confirm the differential lipids, orthogonal partial least square analysis (OPLS-DA) was used and the model parameters were: R^2^X = 0.575, R^2^Y = 0.967, and Q^2^ = 0.602. PCA and OPLS-DA revealed a satisfying distinction between the SR group and the AF group, with obvious separation of samples from different groups (Figure 1A). Based on the results of OPLS-DA, differential lipids were identified as the variables with *p* < 0.05 and variable importance in projection (VIP) > 1 (Table 3). Analysis of lipid classes revealed the different consistency of lipids in the SR group and the AF group (Figures 1B, C). It was noticed that PE and phosphatidylserine (PS) were of higher proportion in the AF group while other distinct lipids were higher in the SR group. Similar to PE, PS is also an important component of mammalian cell membranes, correlated with signal transduction system and development of cell death (Leventis and Grinstein, 2010). The metabolism of these two phospholipids is interrelated. In mitochondria, PS is an essential precursor of PE which is transformed into PE by the enzyme PS decarboxylase (Schuiki and Daum, 2009). The observed high proportion of PE and PS in the AF group indicated the active lipid metabolism in mitochondria. To discover the distinction in precise lipids between the two groups, we summarized the upregulated and downregulated lipids and visualized them with a heat map (Figure 1D). The results showed that PE (16:0p_22:5) and SPH(d22:1) increased in the AF group, with other differential lipid species decreasing such as ceramides (Cer), phosphatidylcholine (PC), and sphingomyelin (SM). Interestingly, PC can be synthesized from PE through methylation in cell (Vance, 2008). This enlightened us that PE might act as an important factor in the abnormal lipid metabolism in AF patients.

**TABLE 2:** Baseline characteristics of all participants.

	AF group (n = 6)	SR group (n = 6)
Age (year)	63.5 ± 3.8	43.5 ± 7.0
Male, n (%)	5 (83.3%)	5 (83.3%)
BMI (kg/m^2^)	22.3 ± 3.5	21.9 ± 2.3
Total cholesterol (mmol/L)	4.6 ± 2.3	3.7 ± 2.1
Triglycerides (mmol/L)	0.8 ± 0.2	0.6 ± 0.3
LDL-C (mmol/L)	3.2 ± 2.1	2.7 ± 1.9
ALT (U/L)	28.2 ± 33.5	30.2 ± 24.8
AST (U/L)	32.7 ± 30.6	33.6 ± 31.1
CR (μmol/L)	97.3 ± 25.2	84.2 ± 14.1

Data were expressed as mean (standard deviation) and numbers (percentages).

AF, atrial fibrillation; SR, sinus rhythm; BMI, body mass index; LDL-C, low-density lipoprotein cholesterol; ALT, alanine aminotransferase; AST, aspartate aminotransferase; CR, creatinine.

**TABLE 3:** Differential lipids in SR and AF group based on orthogonal partial least square analysis (OPLS-DA).

Class	Lipidlon	VIP value	Fold change (AF/SR)	*p*-value
SPH	SPH(d22:1)	1.468	1.332	0.049
PE	PE (16:0p_22:5)	2.221	1.689	0.038
Cer	Cer(d18:1_22:0)	2.454	0.371	<0.001
	Cer(d18:2_24:0)	2.319	0.279	0.003
	Cer(d18:1_23:0)	2.209	0.333	<0.001
	Cer(m42:2 + O)	2.135	0.440	0.003
SM	SM(d36:2)	1.424	0.563	0.012
	SM(d39:1)	1.755	0.389	0.003
PC	PC(17:0_18:2)	1.417	0.589	0.026
	PC(16:1e_18:2)	1.732	0.395	0.005

*p* values were calculated using the *t*-test, with significance set at *p* < 0.05.

Abbreviations: VIP, variable importance in OPLS-DA; AF, atrial fibrillation; SR, sinus rhythm; SPH, sphingosine; PE, phosphatidylethanolamine; Cer, ceramide; SM, sphingomyelin; PC, phosphatidylcholine

### 3.2 PE supplementation aggravates Ang II-induced atrial fibrosis in mice

**FIGURE 2:**
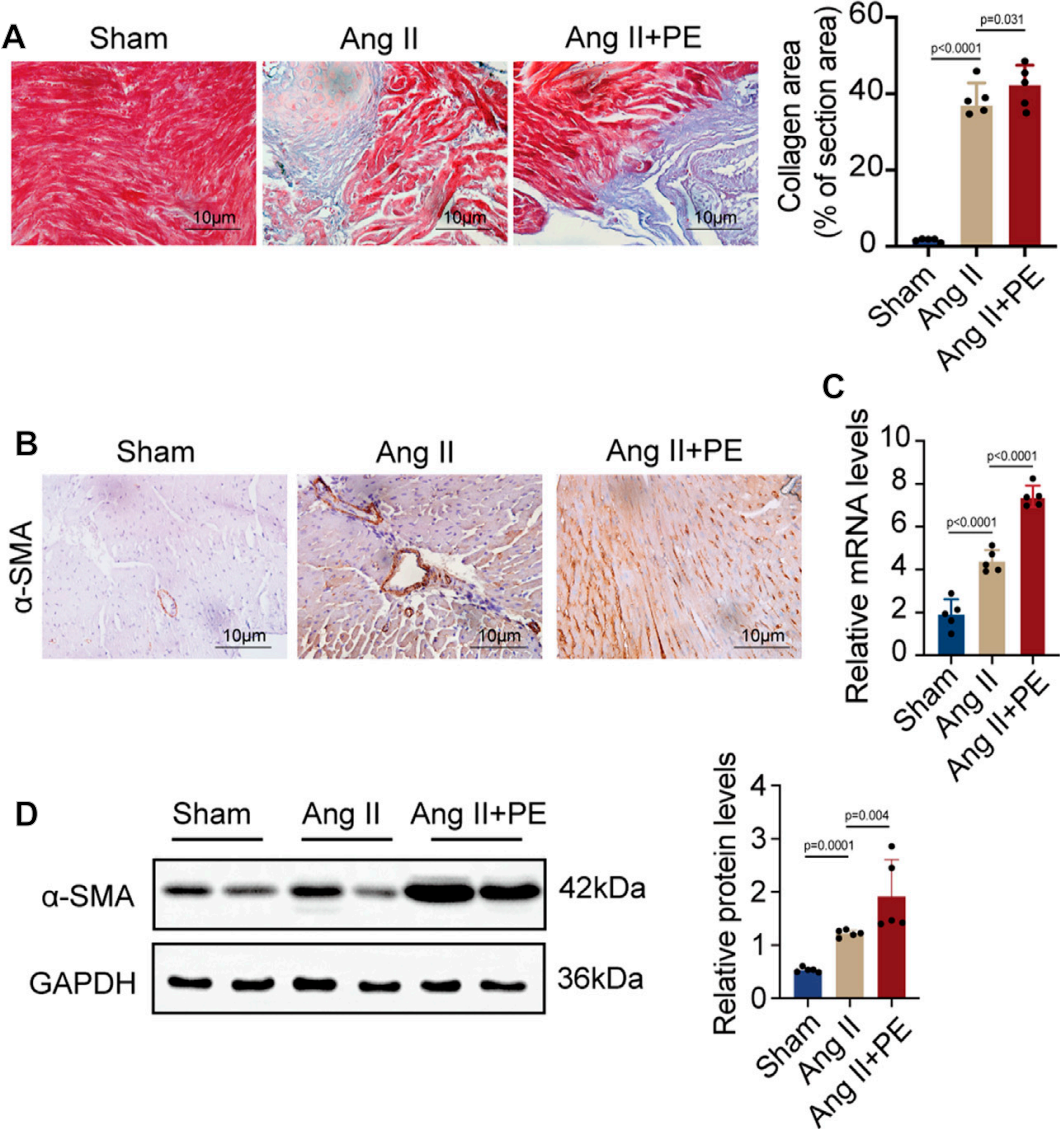
PE aggravates atrial fibrosis in mice induced by Ang II. **(A)** Collagen fiber deposition of atrial tissues was detected by Masson staining (n = 5). Magnification × 40, scale bar 10 µm. **(B)** Representative immunochemistry images for *α*-SMA in the atrial tissues obtained from each group (n = 5). Magnification ×40, scale bar 10 µm. **(C)** Effect of PE on mRNA expression of *α*-SMA in atrial tissues (n = 5). **(D)** Protein expression of *α*-SMA in atrial tissues was detected by Western blot (n = 5). The results were presented as the mean ± SD; *p* < 0.05 was regarded as statistically significant.

AF and atrial fibrosis are intertwined, as AF will induce atrial remodeling by aggravating atrial fibrosis while atrial fibrosis produces substrates to promote AF (Sohns and Marrouche, 2020). It is demonstrated that atrial fibrosis slows the conduction of localized regions and increases the heterogeneity of conduction (Li et al., 1999). Hence, it is crucial to prevent atrial fibrosis from developing and aggravating in order to stop the progression of AF and even reverse the remodeling. Given that previous studies and the results above showed the possible correlation between AF and PE, whether PE plays a role in the development of atrial fibrosis in AF caused our concern. As plenty of evidence has shown that elevated Ang II concentration will increase the occurrence of atrial fibrosis and AF (Goette et al., 2000), we induced atrial fibrosis in mice with Ang II intraperitoneal injection and detected the effect of dietary PE supplementation to explore the impact of PE on atrial fibrosis. After 4 weeks of 2 mg/kg Ang II injection, the atrial fibrosis in mice had aggravated with the significantly larger region of collagen fibers observed (sham group vs. Ang II group, *p* < 0.0001) (Figure 2A). The combination of Ang II and PE supplementation increased the collagen area (Ang II group vs. Ang II + PE group, *p* = 0.031) (Figure 2A). The expression of the fibrosis marker, *α*-SMA, indicated that PE supplement worsened the atrial fibrosis induced by Ang II (Figure 2B). Both the mRNA and protein levels of *α*-SMA were significantly increased in the Ang II group and Ang II + PE group, which revealed the promoting effect of PE on Ang II-induced atrial fibrosis (Figures 2C, D).

### 3.3 PE promotes Ang II-induced ROS generation and cell death in cardiomyocytes

**FIGURE 3:**
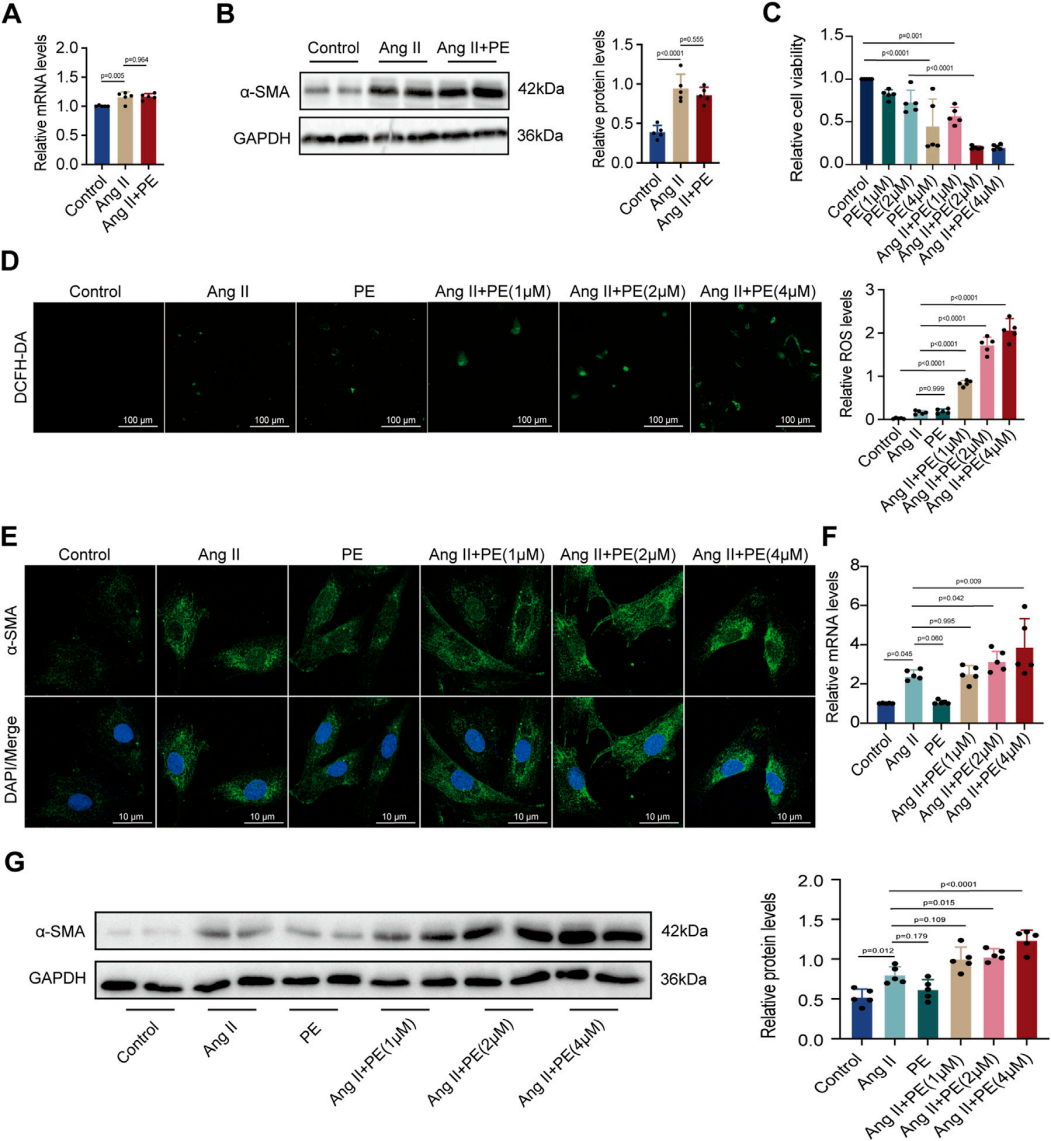
PE increases ROS level and fibrosis in cardiomyocytes instead of atrial fibroblasts induced by Ang II. **(A)** Effect of PE on mRNA expression of *α*-SMA in atrial fibroblasts (n = 5). **(B)** Protein expression of *α*-SMA in atrial fibroblasts was detected by Western blot (n = 5). **(C)** Cardiomyocyte viability was assessed by CCK8 assay (n = 5). **(D)** Representative images of ROS deposition detected by DCFH-DA in cardiomyocytes (n = 5). Magnification ×20, scale bar 100 µm. **(E)** Representative immunofluorescence images for *α*-SMA in each group (n = 5). Magnification ×40, scale bar 10 µm. **(F)** Effect of PE on mRNA expression of *α*-SMA in cardiomyocytes (n = 5). **(G)** Protein expression of *α*-SMA in cardiomyocytes was detected by Western blot (n = 5). The results were presented as the mean ± SD; *p* < 0.05 was regarded as statistically significant.

Given that the remodeling of extracellular matrix mediated by atrial fibroblasts is the major symbol of atrial remodeling, atrial fibroblasts are significant to the maintenance and progression of AF (Jalife and Kaur, 2015). Herein, we evaluated whether PE aggravated atrial fibrosis by promoting atrial fibroblast proliferation to initiate collagen accumulation in atrial tissues. We treated atrial fibroblasts with Ang II and PE to discover the expression of *α*-SMA by RT-qPCR and Western blotting. To our surprise, both mRNA and protein levels of *α*-SMA showed no significant difference between the atrial fibroblasts treated with Ang II and Ang II supplemented with PE (Figures 3A, B). The results implied that PE might induce atrial fibrosis by affecting other cardiac cells instead of atrial fibroblasts.

Forestalling cardiomyocyte death is essential to prevent atrial fibrosis as fibrous tissues will replace the regions of dead cardiomyocytes and cause fibrosis (Nattel, 2017). As we found that PE had no significant effect on atrial fibroblast proliferation, we turned to examine the influence of PE and Ang II on cardiomyocytes. We treated cardiomyocytes with 10 μM Ang II and PE at different concentrations for examinations. Cell viability of cardiomyocytes with different treatments was examined. Apparently, a high concentration of PE caused more cardiomyocyte death (*p* < 0.0001) (Figure 3C). Moreover, PE supplementation exacerbated cell death in Ang II-treated cardiomyocytes (Ang II + PE (1 μM) vs. Ang II + PE (2 μM), *p* = 0.004; Ang II + PE (1 μM) vs. Ang II + PE (4 μM), *p* = 0.004) (Figure 3C). Aiming to discover the reason that PE induced cardiomyocyte death, we analyzed the ROS generation in cardiomyocytes. Administrated with Ang II and 2 μM PE, cardiomyocytes generated slightly more ROS compared to the control group (*p* = 0.542 and 0.345, respectively) (Figure 3D). However, the combination of Ang II and PE significantly increased the ROS levels in cardiomyocytes, which was elevated with the rising concentration of PE (Figure 3D). The results suggested that PE supplementation aggravated ROS generation in Ang II-treated cardiomyocytes. Next, we detected the effect of PE supplementation on *α*-SMA in cardiomyocytes. It was observed that PE increased the cytoplasmic accumulation of *α*-SMA in Ang II-treated cardiomyocytes, which was also correlated with the concentration of PE (Figure 3E). We examined the mRNA and protein levels of *α*-SMA in cardiomyocytes with different treatments. RT-qPCR showed that Ang II increased the transcript level of *α*-SMA (*p* = 0.037), while single PE treatment was unable to have the same effect (*p* > 0.999) (Figure 3F). However, the combination of Ang II and PE significantly elevated the level of *α*-SMA transcription which depended on the concentration of PE as well (Figure 3F). Changes in *α*-SMA protein examined by Western blot analysis were similar to the changes in mRNA levels (Figure 3G). These results demonstrated that PE along with Ang II promoted cell death, ROS formation, and the expression of *α*-SMA in cardiomyocytes, while PE alone could not exert the same influence. Also, considering that a high concentration of PE caused low cell viability, we chose 2 μM as the appropriate dose of PE for further examinations.

### 3.4 PE triggers ferroptosis by suppressing GPX4 and increasing ACSL4 in mice

**FIGURE 4:**
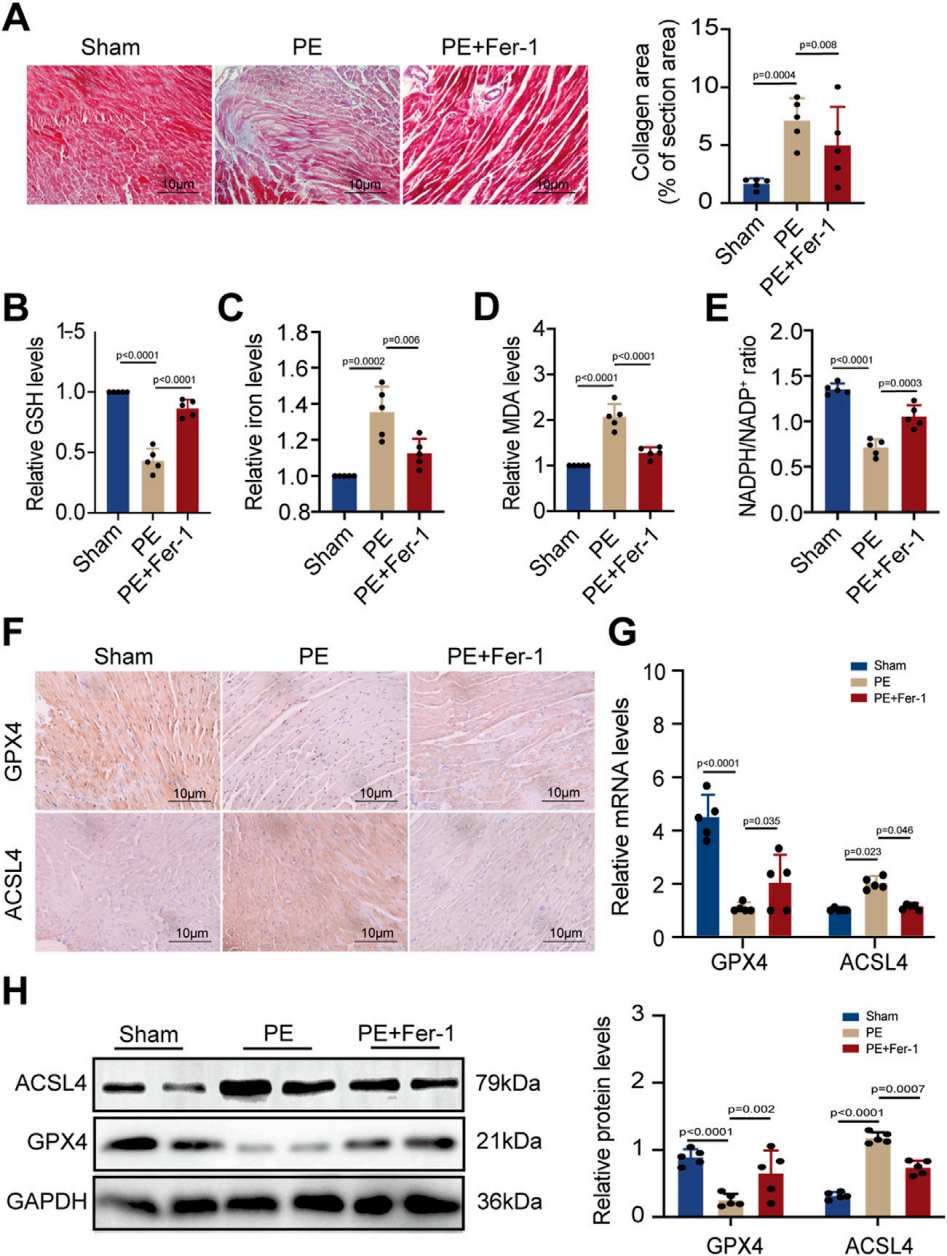
PE induces peroxidation and ferroptosis in atrial tissues. **(A)** Collagen fiber deposition of atrial tissues was detected by Masson staining (n = 5). Magnification ×40, scale bar 10 µm. **(B)** Relative GSH level in atrial tissues. **(C)** Relative iron level in atrial tissues. **(D)** Relative MDA level in atrial tissues. **(E)** NADPH/NADP^+^ ratio in atrial tissues. **(F)** Representative immunochemistry images for GPX4 and ACSL4 in the atrial tissues obtained from each group (n = 5). Magnification ×40, scale bar 10 µm. **(G)** Effect of PE on mRNA expression of GPX4 and ACSL4 in atrial tissues (n = 5). **(H)** Protein expression of GPX4 and ACSL4 in atrial tissues was detected by Western blot (n = 5). The results were presented as the mean ± SD; *p* < 0.05 was regarded as statistically significant.

As PE is a major component of cell membranes, it acts as a crucial part to maintain cell stability. Previous studies have indicated that PE with arachidonic acyl chains is a targeted reaction substrate of lipoxygenase, which oxidizes PE into cytotoxic lipid hydroperoxides that generate excessive ROS and promote ferroptosis (Kagan et al., 2017). The two proteins, GPX4 and ACSL4, are essential in ferroptosis circuitry, with GPX4 deoxidating lipid hydroperoxides and ACSL4 producing sensitive substrates for lipid peroxidation in ferroptosis (Yang et al., 2014; Dixon et al., 2015). Given that PE administration promoted cardiomyocyte death, we detected the effect of dietary PE on mice. Evaluation of collagen area by Masson staining showed that PE increased fibrosis in atrial tissues which could be inhibited by the Fer-1 supplementation (Figure 4A). These results illustrated the potential correlation between ferroptosis and atrial fibrosis caused by PE. To confirm the correlation, we examined the GSH and iron levels in atrial tissues from each group. The GSH level of PE-treated atrial tissues significantly decreased compared to that of the Sham group and PE supplemented with Fer-1 group (Figure 4B). On the contrary, the iron level of PE-treated atrial tissues was the highest among all groups (Figure 4C). As MDA is an important product of lipid peroxidation, we also examined the MDA level in atrial tissues. It was shown that PE improved the production of MDA in atrial tissues, which was decreased by the Fer-1 administration (Figure 4D). NADPH is an essential reductant in cell, which plays an important role in inhibiting ferroptosis by generating cysteine, glutathione, and lipophilic antioxidants (Ding et al., 2020). As a result, the NADPH level exhibits cellular sensitivity to peroxidation and ferroptosis. In the PE-treated mice, the NADPH/NADP^+^ ratio in atrial tissues was suppressed whereas Fer-1 reversed the suppression (Figure 4E). These results suggested that PE initiated ferroptosis and peroxidation in atrial tissues and resulted in fibrosis. To explore possible effectors of PE, we detected the expression of GPX4 and ACSL4 in atrial tissues by IHC assays. It was shown that PE reduced the expression of GPX4 but improved the expression of ACSL4, which could be reversed by Fer-1 administration (Figure 4F). On the basis of the section results, we measured the expression of GPX4 and ACSL4 in atrial tissues by RT-qPCR and Western blotting. The GPX4 transcription decreased significantly while ACSL transcription increased in PE-treated tissues (Figure 4G). But the Fer-1 ameliorated the negative effect of PE on GPX4 and reduced the transcript level of ACSL4 (Figure 4G). Protein levels of GPX4 and ACSL4 shared the same trends with the mRNA levels (Figure 4H), which suggested that PE triggered ferroptosis in atrial tissues by reducing GPX4 and increasing ACSL4.

### 3.5 PE increases ROS formation and induces ferroptosis in cardiomyocytes

**FIGURE 5:**
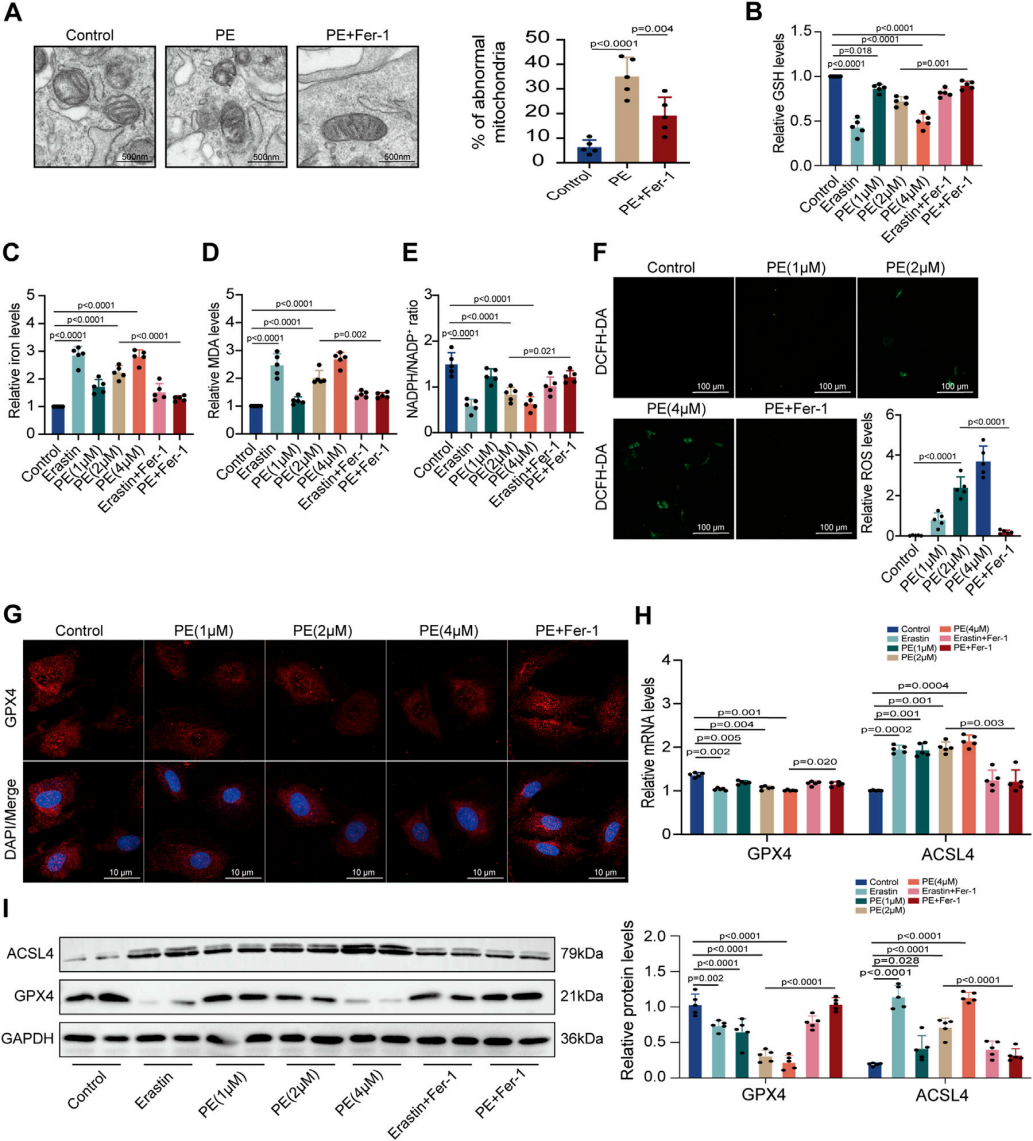
PE initiates ferroptosis by regulating related oxidoreductases in cardiomyocytes. **(A)** TEM observed mitochondrial morphology in cardiomyocytes. The percentage of abnormal mitochondria per image was quantified. Magnification ×7, scale bar 500 nm. **(B)** Relative GSH level in cardiomyocytes. **(C)** Relative iron level in cardiomyocytes. **(D)** Relative MDA level in cardiomyocytes. **(E)** NADPH/NADP^+^ ratio in cardiomyocytes. **(F)** Representative images of ROS deposition detected by DCFH-DA in cardiomyocytes (n = 5). Magnification ×20, scale bar 100 µm. **(G)** Representative immunofluorescence images for GPX4 in each group (n = 5). Magnification ×40, scale bar 10 µm. **(H)** Effect of PE on mRNA expression of GPX4 and ACSL4 in cardiomyocytes (n = 5). **(I)** Protein expression of GPX4 and ACSL4 in cardiomyocytes was detected by Western blot (n = 5). The results were presented as the mean ± SD; *p* < 0.05 was regarded as statistically significant.

To confirm the effect of PE on ferroptosis, we applied TEM to observe the subcellular structure of cardiomyocytes. The normal mitochondria in cardiomyocytes had a complete morphology while shrunken mitochondria with increased membrane density and degeneration of cristae were observed in PE-treated cardiomyocytes. The increased amounts of abnormal mitochondria in cardiomyocytes induced by PE indicated that PE triggered ferroptosis and led to mitochondrial dysfunction in cardiomyocytes (Figure 5A). This was validated by the result that Fer-1 reversed the distortional mitochondria caused by PE (Figure 5A). Next, we detected the GSH, iron, MDA, and NADPH levels of each group with different treatments. It was shown that PE had the same influence on the production of these materials as erastin, a ferroptosis inducer, with GSH and NADPH reducing but iron and MDA increasing (Figures 5B–E). Moreover, the reduction of GSH and NADPH as well as the elevation of iron and MDA depended on the concentration of PE, which was reversed by Fer-1 as that of erastin (Figures 5B–E). ROS levels were examined to determine the effect of PE on intracellular ROS generation. Apparently, PE took part in the activation of ROS formation, with PE concentration related to ROS level (Figure 5F). To illustrate whether PE exerted an influence on proteins concerned with ferroptosis, we used immunofluorescence staining to detect GPX4 in cardiomyocytes. In PE-treated cardiomyocytes, decreased cytoplasmic levels of GPX4 correlated with the high concentration of PE were observed, which were improved by the Fer-1 administration (*p* < 0.0001) (Figure 5G). Meanwhile, the transcription of GPX4 and ACSL4 varied with the PE dose. The transcript level of GPX4 was lower when cardiomyocytes were treated with a high concentration of PE, while the transcript level of ACSL4 acted oppositely in the same condition (Figure 5H). Protein levels of GPX4 and ACSL4 were mostly consistent with the mRNA levels (Figure 5I). The results indicated that PE had the same effect as erastin on inducing ferroptosis in cardiomyocytes, which could all be inhibited by Fer-1.

### 3.6 Ferroptosis played an important role in the atrial fibrosis induced by both single Ang II and Ang II supplemented with PE

**FIGURE 6:**
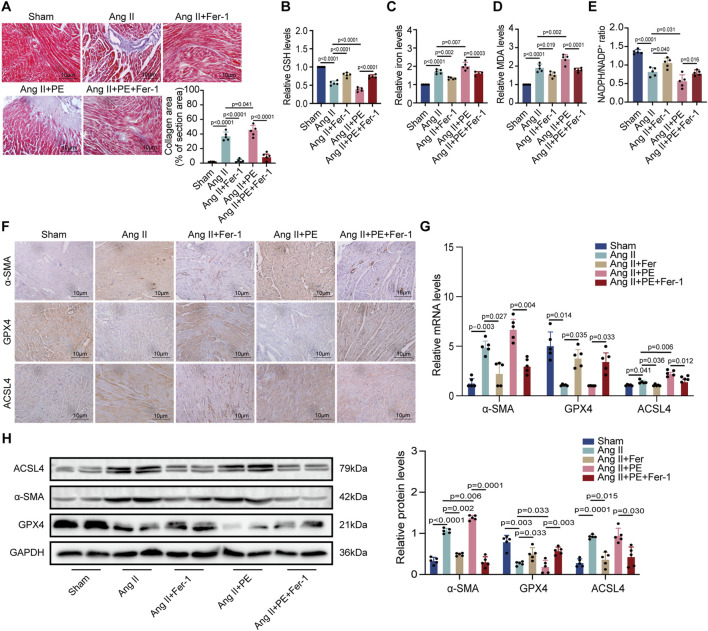
PE promotes AngII-induced atrial fibrosis by initiating ferroptosis. **(A)** Collagen fiber deposition of atrial tissues was detected by Masson staining (n = 5). Magnification ×40, scale bar 10 µm. **(B)** Relative GSH level in atrial tissues. **(C)** Relative iron level in atrial tissues. **(D)** Relative MDA level in atrial tissues. **(E)** NADPH/NADP^+^ ratio in atrial tissues. **(F)** Representative immunochemistry images for *α*-SMA, GPX4, and ACSL4 in the atrial tissues obtained from each group (n = 5). Magnification ×40, scale bar 10 µm. **(G)** Effect of PE on mRNA expression of *α*-SMA, GPX4, and ACSL4 in atrial tissues (n = 5). **(H)** Protein expression of *α*-SMA, GPX4, and ACSL4 in atrial tissues was detected by Western blot (n = 5). The results were presented as the mean ± SD; *p* < 0.05 was regarded as statistically significant.

To explain whether PE supplementation promoted atrial fibrosis in mice with Ang II administration, we detected the collagen area in atrial tissues with different treatments. Masson staining showed that Fer-1 not only alleviated the fibrosis in atrial tissues treated with Ang II but also inhibited the worse collagenization induced by the combination of Ang II and PE (Figure 6A). Next, we detected the GSH, iron, MDA, and NADPH levels in atrial tissues from each group. It was revealed that Ang II with PE supplementation caused more severe peroxidation and a higher level of iron than a single Ang II administration, which could be eased by Fer-1 (Figures 6B–E). These results illustrated that ferroptosis might act as the key factor in the atrial fibrosis induced by Ang II with PE supplementation. IHC staining confirmed that the increasing *α*-SMA expression induced by Ang II and PE was reduced by Fer-1 (Figure 6F). Moreover, Fer-1 elevated the expression of GPX4 whereas lessened that of ACSL4 in the atrial tissues with Ang II and PE administration (Figure 6F). To confirm the result on a molecular level, we performed RT-qPCR and Western blotting to analyze the related molecules. It was shown that both transcript levels of *α*-SMA and ACSL4 increased significantly while GPX4 transcription decreased in the Ang II group and Ang II + PE group (Figure 6G). However, Fer-1 reversed the upregulation of *α*-SMA and ACSL4 as well as the downregulation of GPX4 (Figure 6G). The protein levels of the related molecules presented the same trends as the mRNA levels (Figure 6H). It was suggested that ferroptosis played an essential part in the atrial fibrosis induced by both single Ang II and Ang II supplemented with PE.

### 3.7 Inhibition of ferroptosis alleviated mitochondrial damage and myocardial fibrosis induced by Ang II and PE in cardiomyocytes

**FIGURE 7:**
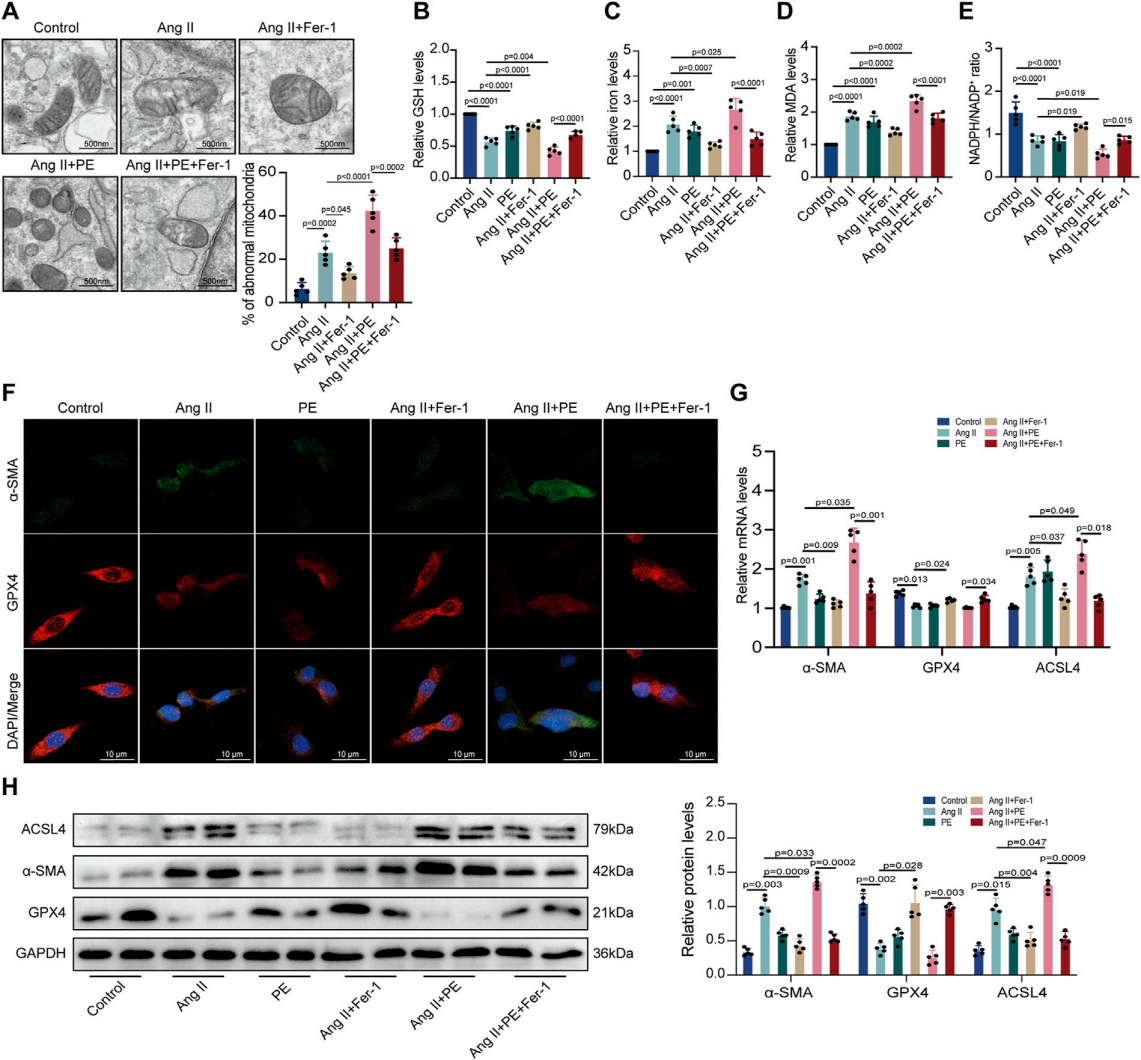
PE induces ferroptosis in Ang II-treated cardiomyocytes. **(A)** TEM observed mitochondrial morphology in cardiomyocytes. The percentage of abnormal mitochondria per image was quantified. Magnification ×7, scale bar 500 nm. **(B)** Relative GSH level in cardiomyocytes. **(C)** Relative iron level in cardiomyocytes. **(D)** Relative MDA level in cardiomyocytes. **(E)** NADPH/NADP^+^ ratio in cardiomyocytes. **(F)** Representative immunofluorescence images for *α*-SMA and GPX4 in each group (n = 5). Magnification ×40, scale bar 10 µm. **(G)** Effect of PE on mRNA expression of *α*-SMA, GPX4, and ACSL4 in cardiomyocytes (n = 5). **(H)** Protein expression of *α*-SMA, GPX4, and ACSL4 in cardiomyocytes was detected by Western blot (n = 5). The results were presented as the mean ± SD; *p* < 0.05 was regarded as statistically significant.

Then, we analyzed the impact of PE on ferroptosis in cardiomyocytes. TEM observed mitochondrial damage and dysfunction in both Ang II-treated and Ang II + PE-treated cardiomyocytes, which was inhibited by Fer-1 (Figure 7A). This demonstrated that suppressing ferroptosis could ameliorate mitochondrial damage caused by Ang II and PE in cardiomyocytes. Then we detected the GSH, iron, MDA, and NADPH levels in cardiomyocytes with different treatments. Both Ang II and Ang II combined with PE significantly reduced the GSH and NADPH levels but elevated the iron and MDA levels in cardiomyocytes (Figures 7B–E). Nevertheless, this could be reversed by Fer-1 administration (Figures 7B–E), which further explained that ferroptosis was essential to intracellular dysfunction caused by Ang II and PE. Immunofluorescence staining showed that Fer-1 meliorated the increase of *α*-SMA and the reduction of GPX4 induced by Ang II and PE in cardiomyocytes (Figure 7F). To quantify the change of Ang II and PE on the expression level, mRNA and protein levels in cardiomyocytes were examined. The transcript level of *α*-SMA was significantly higher in the cardiomyocytes treated with Ang II and Ang II supplemented with PE, which was downregulated by Fer-1 (Figure 7G). Meanwhile, Fer-1 upregulated the GPX4 transcription but reduced the ACSL4 transcription, which was opposite to the effect of Ang II and PE in cardiomyocytes (Figure 7G). Consistent with the mRNA level, the expression of *α*-SMA, GPX4, and ACSL4 proteins indicated that inhibition of ferroptosis alleviated myocardial fibrosis induced by Ang II and PE in cardiomyocytes (Figure 7H).

## 4 Discussion

**FIGURE 8:**
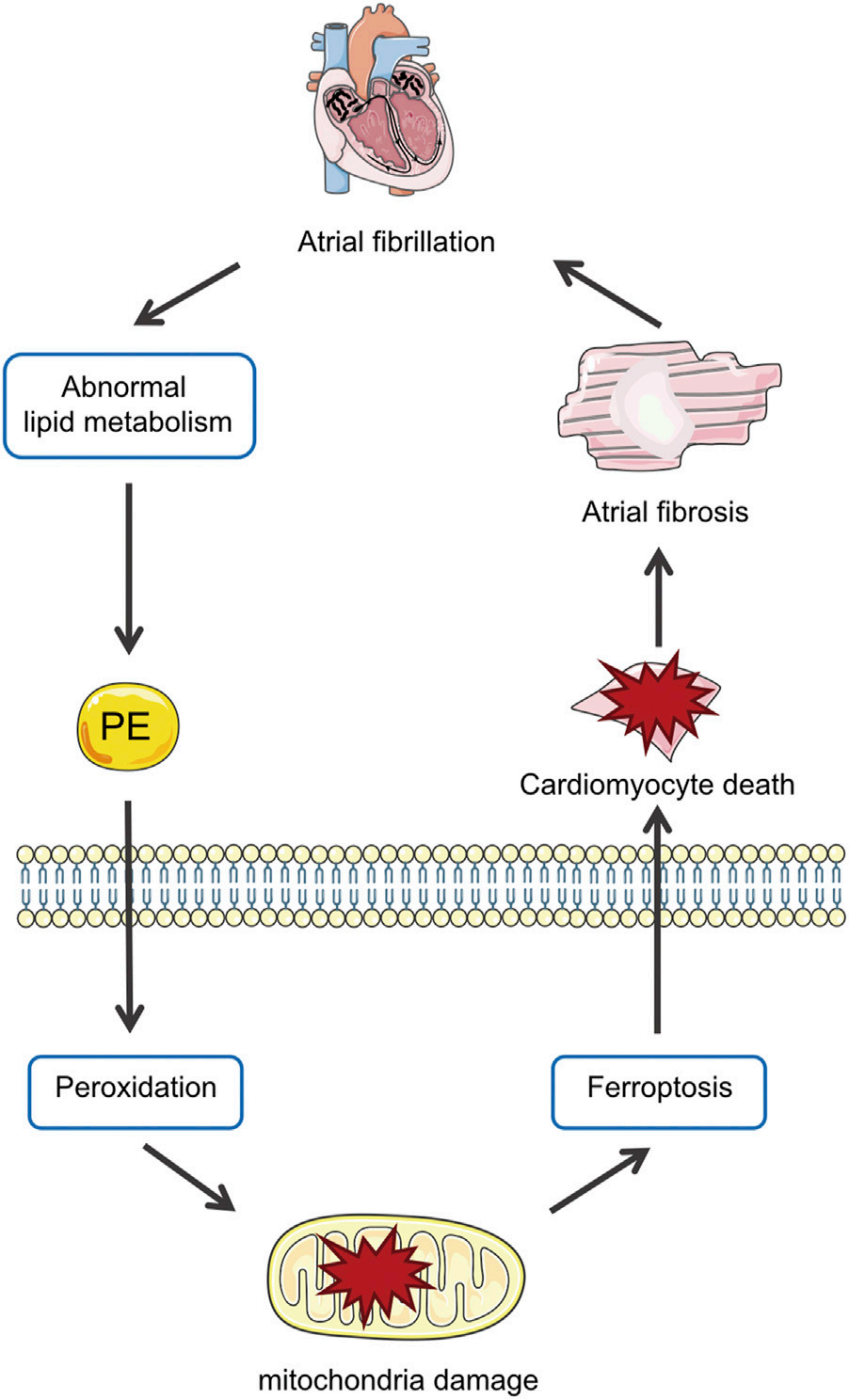
Abnormal metabolism in AF increases PE in the myocardium, which promotes peroxidation and thus ferroptosis in cardiomyocytes that aggravate atrial fibrosis by causing mitochondria damage and cell death.

The main results of the present study are as follows: 1) Lipidomics demonstrated the lipid profiles in patients with AF and identified PE as the differential lipid associated with AF; 2) In Ang II-induced mice, PE supplementation aggravated atrial fibrosis and increased peroxidation which could be alleviated by a ferroptosis inhibitor; 3) PE affected cardiomyocytes by promoting oxidation products and mitochondrial damage, which aggravated cardiomyocyte death induced by Ang II; 4) PE triggered ferroptosis in cardiomyocytes to participate in myocardium fibrosis (Figure 8).

Though abundant researches have explained various mechanisms of AF, effective treatment to prevent AF from progressing remains to be developed (Nattel et al., 2020). Notably, lipid metabolism is reported to be closely correlated with AF. A large and nationwide study with almost 14,000 participants in Poland demonstrated the inverse associations between lipid levels and AF (Harrison et al., 2020). To further clarify the correlation between lipid species and AF, researchers apply lipidomics to the metabolic analysis of AF (Zhou et al., 2019). Compared with normal people, AF patients have significantly different lipid profiles and several lipid species can serve as the predictive factors of AF (Del Greco M et al., 2019; Tham et al., 2020). Zhou et al. discovered that fatty acid metabolism was perturbed by AF and specific fatty acids showed a linear correlation with the left atrial area. A study based on 3,772 participants in the ADVANCE trial examined 6 kinds of lipids associated with AF prevalence and confirmed the predictive value of the model based on these lipids in mice model (Tham et al., 2021). In the present study, we also applied lipidomics based on UHPLC-MS/MS to attain the lipid profiles of AF. In PCA and OPLS-DA plots, AF patients were distinguished from healthy controls and differential lipids were screened out. Specifically, we found that PE was the most significantly upregulated lipid in AF patients among the differential lipids. Moreover, we observed the elevation of PS in AF patients. As PE and PS are two major phospholipids in organism that can transform into each other, especially in mitochondria, this suggests a specific pattern of phospholipid metabolism and active mitochondrial metabolism in AF patients.

The potential mechanisms of AF include electrical and structural remodeling, dysfunction of autonomic nervous system dysfunction, and abnormal calcium homeostasis (Li et al., 2021a). Among these, structural remodeling plays a pivotal role in linking all the mechanisms, which can affect other mechanisms by reconstructing the component of myocardium and interrupting the conduction of electrical signals and ion channels (Yue et al., 2011; Rog-Zielinska et al., 2016). As a result, the progression of AF is vastly associated with atrial fibrosis. Several clinical trials demonstrated timely intervention of atrial fibrosis may prevent or even reverse the progression of AF. Cochet et al. discovered that left atrial dysfunction is closely associated with scar burden in patients with persistent AF even years after successful ablation (Cochet et al., 2014). It is also reported that AF patients with heart failure present with more severe atrial fibrosis from late gadolinium enhancement cardiac magnetic resonance imaging (Akkaya et al., 2013). Moreover, animal experiments have indicated that atrial fibrosis not only improves conduction heterogeneity but also disturbs conduction, which delays and blocks electrical waves in the atrial wall (Velagapudi et al., 2013). Atrial fibrosis is a process that myocardial geometry is regulated to accommodate new physiological conditions and minimize the influences of mechanical, chemical, and electrical stimuli (Dzeshka et al., 2015). It presents as the proliferation and differentiation of fibroblasts and the replacement of cardiomyocytes that can conduct normal electric signals (Burstein and Nattel, 2008). As no valid evidence indicates purely interstitial fibrosis can damage longitudinal conduction, it is suggested to prevent cardiomyocyte death rather than inhibiting fibroblast proliferation. In fact, in addition to being initiated by fibroblast activation, atrial fibrosis can also take place when extensive cardiomyocyte death causes the interruption of cardiac muscle bundles (Weber et al., 2013). Cardiomyocyte loss in atrium can recruit myofibroblasts to produce autocrine/paracrine factors and profibrotic signals (Tarone et al., 2014). This will promote collagen accumulation and result in cardiomyocyte hypertrophy (Dzeshka et al., 2015). In our study, with the discovery of PE elevation in AF patients, we administrated PE in Ang II-induced models *in vivo* and *in vitro* to explore whether PE exerted an impact on atrial fibrosis in AF. We found that PE promoted the atrial fibrosis induced by Ang II, which demonstrated that abnormal phospholipid metabolism might aggravate atrial fibrosis by overproducing PE in AF patients. Moreover, we discovered that PE induced atrial fibrosis by affecting cardiomyocytes rather than atrial fibroblasts. This might illustrate that PE targeted cardiomyocytes but exerted no distinct influence on the activation of myofibroblasts. As the major energy source of the heart relies on the oxidation of lipids, abnormal lipid uptake and oxidation will affect cardiomyocytes and eventually result in cardiomyopathy (Goldberg et al., 2012). Based on our results, we deduced that PE elevation disturbed the metabolic balance of lipids in the heart, which mostly influenced cardiomyocytes by causing mitochondrial dysfunction and cell death. Interestingly, Jang et al. discovered analogous results that accumulation of peroxidized PE and disruption of mitochondria are features of ferroptosis in cardiomyocytes (Jang et al., 2021). Altogether, these revealed that PE targeted cardiomyocytes and promoted ferroptosis in Ang II-induced atrial fibrosis.

As PE is an abundant component of cell membrane, it is essential for multiple cellular processes. The activity of PE affects the integrity of mitochondrial morphology and therefore influences mitochondrial functions, which are correlated with cardiovascular diseases (Colina-Tenorio et al., 2020). In a lipidomics study, PE was found higher in patients at high risk of cardiovascular disease, which could be a potential biomarker to evaluate cardiovascular health status (Rivas Serna et al., 2021). Also, oxidation of PE was discovered to be associated with stable angina, coronary heart disease, and cerebrovascular diseases due to inflammation or PCD caused by PE (Zhong et al., 2019). Specifically, a study applying quantitative redox lipidomics, reverse genetics, bioinformatics, and systems biology revealed AA/AdA-containing species of PE as the ferroptosis signals produced in the endoplasmic reticulum (Kagan et al., 2017). Furthermore, GPX4 participated in PE-related ferroptosis as the insufficiency of GPX4 triggered lipid metabolism that overproduced PE containing AA/AdA (Kagan et al., 2017). They also discovered that ACSL4 might take part in the AA/AdA esterification into PE and promote PE-navigated ferroptosis (Kagan et al., 2017). Herein, we examined whether ferroptosis could be triggered by PE in myocardium. As GSH depletion, lipid ROS accumulation, and iron accumulation are characteristics of ferroptosis (Fang et al., 2019), we found that PE administration promoted peroxidation, iron elevation, GSH reduction, and abnormal mitochondrial morphology. Moreover, PE could downregulate GPX4 expression and upregulate ACSL4 expression to induce ferroptosis and cardiomyocyte death. The reverse of the effects above by the ferroptosis inhibitor Fer-1 further confirmed the discovery.

Ferroptosis is a non-apoptotic PCD in which a large amount of cellular iron and lipid hydroperoxide result in overwhelming lipid accumulation and disturb the homeostasis of redox reactions (Stockwell et al., 2017). It causes ROS accumulation and eventually destroys the integrity of cell membrane, especially of mitochondria (Qiu et al., 2020). Therefore, the representative morphology of ferroptosis is shrunken mitochondria, with increased membrane density and the degeneration and breakdown of cristae (Xie et al., 2016). It is reported that glutaminolysis can inhibit ferroptosis by reducing ROS accumulation, with GSH and GPX4 as the pivotal factors (Bentea et al., 2021). Catalyzed by GPX4, GSH converses to glutathione disulfide while lipid hydroperoxides are reduced to lipid hydroxy derivative (Mirochnitchenko et al., 2000). Herein, the inactivation of GPX4 or the depletion of GSH leads to the occurrence of ferroptosis. In the meantime, ACSL4 participates in ferroptosis by acylating AA-containing PE into membrane to promote the sensitivity of lipid peroxidation (Dixon et al., 2015). The role of ferroptosis with abnormal glutaminolysis and ROS overproduction in the pathology of many diseases is extensively studied. Jankauskas et al. found that ferroptosis could be triggered by coronavirus disease 2019, which led to lipid peroxidation in human endothelial cells (Jankauskas et al., 2023). In addition, it has been put forward that inhibiting ferroptosis is a promising method to treat ischemic organ injuries (Stockwell et al., 2020). Notably, cardiomyocytes are extremely sensitive to iron overload (Lakhal-Littleton et al., 2016). High cellular iron concentration will initiate the Fenton reaction to overproduce ROS and cause ferroptosis (Doll and Conrad, 2017). Recent studies have provided evidence that ferroptosis participates in various cardiovascular diseases. In Doxorubicin-induced cardiotoxicity, ferroptosis is reported to promote myocardium damage and cardiomyocyte cell death, which could be ameliorated by an angiotensin receptor neprilysin inhibitor *via* AKT/SIRT3/SOD2 signaling pathway activation (Liu et al., 2022). Furthermore, Zhuang et al. indicated miRNA-375-3p could increase iron levels and downregulate GPX4 to induce mitochondrial dysfunction and increase ROS in myocardial fibrosis after myocardial infarction (Zhuang et al., 2022). These studies reveal that ferroptosis plays an important role in the occurrence and progression of myocardium fibrosis. Ferroptosis is also observed in Ang II-induced models of cardiovascular diseases. It is reported that cystine/glutamate antiporter xCT could promote cystine uptake and thus improve glutaminolysis, which could prevent ROS from overproduction and protect cardiomyocytes from ferroptosis in Ang II-induced cardiac hypertrophy (Zhang et al., 2022a). Moreover, Zhang et al. found that Ang II induced an increase in iron level and lipid peroxidation in myocardium which caused pathological myocardial remodeling and ultrastructural injury in mice (Zhang et al., 2022b). They observed cardiac fibrosis and specific ferroptotic morphology in Ang II-treated mice which were ameliorated by Fer-1 (Zhang et al., 2022b). Similar to these results, we discovered that Ang II increased iron, peroxide, and fibrosis markers but reduced the GPX4, NADPH, and GSH that participate in preventing ferroptosis. Nevertheless, we found Ang II failed to induce fibrosis in atrial fibroblasts but promoted cardiomyocyte hypertrophy and death instead. These results implied that cardiomyocytes were mainly affected by Ang II and presented with lipid peroxidation and mitochondrial damage caused by ferroptosis. Moreover, the supplementation of PE enhanced the cardiac injury caused by Ang II, which could also be eased by the ferroptosis inhibitor. According to the results of the present study, PE exerts a negative influence on cardiomyocytes by triggering ferroptosis and causing cell death. What’s more, PE might aggravate the atrial fibrosis induced by Ang II, which indicates that PE might play an important role in atrial remodeling in AF progression.

Interestingly, ferroptosis also occurs in other pathological models induced by Ang II. Li et al. reported that Ang II infusion could trigger renal ferroptosis, epithelial-mesenchymal transition and interstitial fibrosis, which was regulated by Nrf2/xCT/GPX4 pathway (Li et al., 2022). In Ang II-induced astrocytes, downregulation of GPX4 and oxidative stress were observed which could be inhibited by Fer-1 and the maintenance of Nrf2/Keap1/HO-1 signaling pathway (Li et al., 2021b). In our study, Ang II infusion also resulted in the reduced expression of GPX4 and led to ferroptosis in cardiomyocytes. As GPX4 is the key enzyme in GSH conversion to exert the antioxidative effect, it is believed that Ang II can promote ROS generation by downregulating GPX4 and disturbing glutaminolysis. In general, the effect of Ang II on the activation of immune cells and the promotion of inflammation has been discussed by many researches (Yuan et al., 2022). But the influence of RAS and its effectors on redox imbalance needs more attention. In the present study, it is noted that Ang II also led to the increased expression of ACSL4, which is essential to lipid decomposition and utilization. This suggested that Ang II might promote oxidative stress by affecting both glutaminolysis pathway and lipid peroxidation, which eventually results in susceptibility to ferroptosis. Moreover, with PE supplement, cellular phospholipids were shaped and lipid peroxidation was activated, which promoted ferroptosis and caused membrane damage in mitochondria. These findings revealed the role of RAS and phospholipid metabolism in cellular redox balance.

It is reported that myocardial energy metabolism is vital in the atrial structural remodeling of AF (Kim et al., 2014). Given that fatty acids are the main metabolites of the heart, the maintenance of cardiac structure and function is dependent on lipid metabolism (Sharma et al., 2004). Hence, abnormal lipid metabolism might lead to the overproduction of metabolites and subsequently myocardial dysfunction. This is further confirmed by a recent study that showed four genes, *ITGB1*, *HSP90AA1*, *CCND1*, and *HSPA8*, regulate lipid metabolism to promote the occurrence of AF (Fang et al., 2022). In the present study, we found distinct lipid profiles of AF patients, with PE as the differential upregulating lipid. Our data also showed that the inhibition of ferroptosis triggered by PE could alleviate Ang II-induced atrial fibrosis. The discovery might offer a new perspective to focus on the effect of lipid metabolism on atrial fibrosis and the progression of AF. Moreover, the results also suggest that restraint of cardiomyocyte ferroptosis might be a potential therapy for atrial remodeling.

Our study suffers from some limitations. Although the OPLS-DA score presented distinguishable lipid profiles of AF with satisfying model parameters, larger sample sizes and paired participants are still required to confirm the discovery. Moreover, the indicators of atrial fibrosis examined in the present study were insufficient. More examinations should be carried out for further validation. As PE is a multifunctional molecule in cell, there might be other mechanisms aside from ferroptosis through which PE can affect atrial fibrosis. Also, the *in vivo* bioavailability of PE was not detected in the present study. Further studies are required to explore the effect of PE on the progression of AF.

## 5 Conclusion

In summary, we demonstrated the differential lipid profiles of AF patients and revealed the potential effect of PE on atrial remodeling. We found that PE promoted Ang II-induced atrial fibrosis by increasing oxidation products and regulating related oxidoreductases, leading to mitochondrial damage and ferroptosis in cardiomyocytes. Our findings suggest that inhibition of PE and ferroptosis can serve as potential therapy to prevent AF progression.

## Data Availability

The original contributions presented in the study are included in the article/supplementary material, further inquiries can be directed to the corresponding authors.
